# The Spectrum of Uncommon Adverse Effects in Patients Treated With Levetiracetam

**DOI:** 10.7759/cureus.104638

**Published:** 2026-03-03

**Authors:** Jayaram Saibaba, Murugesan Subramaniam, Arunprakash Thangavelu, Krithikaa Chitharanjan Rennukaranjan

**Affiliations:** 1 Neurology, Mahatma Gandhi Medical College and Research Institute, Puducherry, IND; 2 Medicine, Mahatma Gandhi Medical College and Research Institute, Puducherry, IND

**Keywords:** adverse drug reactions, antiseizure medications, epilepsy, levetiracetam, neuropsychiatric effects, pharmacovigilance

## Abstract

Background: Levetiracetam is a commonly prescribed broad-spectrum antiseizure medication (ASM) known for its effectiveness and favourable pharmacokinetic profile, leading to its widespread adoption as first-line therapy for focal and generalized epilepsy worldwide. While its well-known side effects are documented, a range of unusual and serious psychiatric and physical side adverse drug reactions (ADRs) is becoming more recognized but less measured.

Objective: To present 15 patients who experienced unusual adverse effects linked to levetiracetam, examining demographic patterns, dose relationships, and management outcomes in a real-world clinical setting.

Methods: We reviewed 15 consecutive cases from Mahatma Gandhi Medical College and Research Institute (MGMCRI) hospital in Puducherry, India, where an unusual adverse effect occurred during levetiracetam monotherapy or combination therapy between 2020 and 2024. We collected and analysed data on patient demographics, levetiracetam dose, descriptions of adverse effects, and concomitant ASMs. We also retrospectively applied a standardized causality assessment (Naranjo ADR probability scale). Data analysis was performed using SPSS version 26.0 (IBM Corp., Armonk, NY, USA)

Results: The mean age of the cohort was 43.1 years (standard deviation [SD] ± 19.6 years), with a male predominance (9/15, 60%). The mean dose of levetiracetam before patients reported experiencing adverse effects was 1400 mg/day (SD ± 450 mg/day; range: 500-2000 mg/day). The mean time from levetiracetam initiation or dose adjustment to adverse effect onset was 30 days (range: 7-90 days), providing a clinically useful window for heightened monitoring. Neuropsychiatric adverse effects predominated (12/15, 80%), including behavioural dysregulation (hypersexuality, over-spirituality, akathisia), mood disorders (severe depression, suicidal tendency, mania), anxiety-related symptoms (claustrophobia, insomnia), and cognitive disturbances. Non-neuropsychiatric adverse effects (3/15, 20%) included excessive bleeding, difficulty in urination, and weight gain. Naranjo scale scores ranged from 5 to 8, indicating probable causality in all cases. The most common management approach involved medication adjustment - either levetiracetam dose reduction or switching to lacosamide, sodium valproate, or brivaracetam - with adverse effects resolving approximately three to four months after therapeutic modification, often with adjunctive psychiatric intervention.

Conclusion: Early recognition of these uncommon neuropsychiatric and non-neuropsychiatric adverse effects, beyond the standard somnolence or irritability, is critical for patient safety and medication adherence. Recognition of these neurological adverse effects and appropriate therapy changes will help ensure patient safety, satisfaction with drug therapy, and adherence to the prescribed regimen. Clinicians should maintain heightened vigilance for atypical presentations following levetiracetam initiation. Further research should be conducted to determine the true incidence and risk factors associated with these distressing adverse effects, as the limitations of this retrospective review do not support definitive conclusions regarding these relationships.

## Introduction

Levetiracetam is a second-generation antiseizure medicine (ASM) that is widely used and well tolerated; therefore, it is considered first-line therapy for both focal and generalised epilepsies worldwide [1]. Its favourable pharmacokinetic profile, including rapid absorption, linear kinetics, minimal protein binding, and lack of hepatic cytochrome P450 metabolism, contributes to its clinical utility and favourable drug-drug interaction profile [2]. Levetiracetam works by modulating synaptic vesicle protein 2A (SV2A), which is different than the way most other antiseizure medicines work [3]. SV2A is a transmembrane glycoprotein involved in synaptic vesicle exocytosis and neurotransmitter release, and its modulation by levetiracetam appears to enhance inhibitory neurotransmission and reduce seizure susceptibility [4]. Commonly reported side effects include somnolence, asthenia, dizziness, and behavioural changes such as irritability or aggression, which are reported in 10-15% of patients [5].

Adverse drug reactions (ADRs) are defined as noxious and unintended responses to a medicinal product occurring at doses normally used in humans, distinguished from 'side effects,' which may include desirable or undesirable effects that are predictable from a drug's pharmacological profile. The prevalence of these neuropsychiatric adverse effects has been well-documented in clinical trials and post-marketing surveillance, with somnolence affecting approximately 14.8% of patients and behavioural symptoms occurring in up to 13.3% of adults and 23.6% of children [6].

However, post-marketing surveillance and case reports have highlighted a spectrum of less frequent but potentially severe and distressing adverse effects. These range from profound neuropsychiatric disturbances, such as severe depression, psychosis, and marked behavioural dysregulation, to uncommon non-neuropsychiatric adverse effects including thrombocytopenia, movement disorders, and gastrointestinal disturbances [7,8]. The reported incidence of psychiatric adverse events requiring intervention ranges from 1-2% in clinical trials but may be substantially higher in real-world clinical settings, particularly among patients with pre-existing psychiatric comorbidities [9].

The true incidence and specific risk factors for these reactions remain poorly characterised in the literature. Proposed risk factors include rapid dose titration, high maintenance doses, history of psychiatric illness, concomitant ASMs with psychotropic effects, and genetic polymorphisms affecting SV2A binding affinity or drug metabolism [10]. This manuscript presents 15 patients exhibiting uncommon adverse effects attributed to levetiracetam, aiming to enhance clinical awareness and reporting. While individual case reports exist, compiled case series are essential for characterising the spectrum of these reactions, identifying potential patterns, and guiding clinical management strategies.

## Materials and methods

Study setting and population

We reviewed the patients seen in our neurology clinic at a tertiary care hospital, Mahatma Gandhi Medical College and Research Institute (MGMCRI) hospital in Puducherry, India, who were treated with levetiracetam from January 2020 to December 2024 and experienced an uncommon ADR secondary to the medication.

Inclusion and exclusion criteria

The inclusion criteria required that patients were treated with levetiracetam, either as monotherapy or combination therapy, for seizure control. Eligible patients must have developed a new, clinically significant symptom that could not be explained by seizure activity, other diseases, or concomitant medications. The symptom must have been determined by the neurologist to have a ‘probable’ or ‘certain’ association with levetiracetam, corresponding to a score greater than five on the Naranjo ADR probability scale. Additionally, the adverse reaction frequency needed to be recorded as at least ‘common’ (>1/100 to <1/10) or ‘very common’ (>1/10), or alternatively classified as ‘uncommon’, ‘rare’, or ‘very rare’ according to standard pharmacological frequency classifications.

Exclusion criteria included incomplete medical records that precluded adequate assessment, as well as the presence of an alternative explanation for the symptoms, such as ongoing seizure activity or intercurrent illness. Patients were also excluded if the Naranjo scale score was less than 5, indicating a possible or doubtful association.

Sampling method and sample size

This was a retrospective, descriptive case series. All patients meeting the predefined inclusion criteria during the five-year study period (2020-2024) were included in the analysis. During this period, approximately 4,800 patients with epilepsy were evaluated in our neurology clinic, of whom approximately 1,200 (25%) received levetiracetam as either monotherapy or combination therapy. The 15 cases presented represent all identified patients with uncommon levetiracetam-associated ADRs meeting our inclusion criteria during this period. In all 15 patients, baseline blood investigations done before starting the drug (complete hemogram, renal and liver function test, coagulation profile, prothrombin time, bleeding and clotting time) were normal. While we attempted to identify all eligible cases through a systematic review of medical records, the retrospective design precludes certainty that all cases were captured, representing a potential selection bias.

Given the exploratory and descriptive nature and because the adverse effects under investigation are uncommon (estimated incidence < 1-5% based on literature), no formal sample size calculation was performed, as this study aimed to describe the spectrum of observed adverse effects rather than estimate population parameters with predetermined precision.

Denominator data

Our neurology clinic evaluates approximately 80-100 patients with epilepsy monthly (approximately 1,000-1,200 annually). During the five-year study period, levetiracetam was prescribed to approximately 25% of epilepsy patients (n≈1,200), with the remainder receiving other ASMs, including valproate, phenytoin, carbamazepine, oxcarbazepine, lacosamide, and clobazam. The 15 cases reported represent approximately 1.25% of levetiracetam-treated patients during this period, consistent with the expected low incidence of these unusual adverse effects.

Data collection and variables

Data extracted from the cases included age of the patient, sex of the patient, levetiracetam dose at symptom development, description of adverse reaction, concomitant antiseizure medication(s), time to symptom onset after levetiracetam initiation or dose adjustment, management strategies, and time to symptom resolution after intervention.

Neuropsychiatric adverse effects, including behavioural changes such as hypersexuality and over-spirituality, were identified through clinical interviews with patients and caregivers. Hypersexuality was defined as a marked increase in sexual thoughts, urges, or behaviours that were distressing to the patient or family and represented a clear change from baseline. Over-spirituality was defined as a new or intensively increased preoccupation with religious thoughts, rituals, or behaviours that significantly interfered with daily functioning. These assessments were made by treating neurologists and, where indicated, confirmed by psychiatric consultation. However, no standardized quantitative scales were employed, representing a methodological limitation.

Causality assessment

The Naranjo ADR probability scale was retrospectively applied to each case by two independent neurologists, with disagreements resolved by consensus. The Naranjo scale is a 10-item questionnaire assessing the likelihood that an adverse event is attributable to a specific medication, with scores interpreted as: ≥9 = definite, 5-8 = probable, 1-4 = possible, ≤0 = doubtful. Permission for scale usage is not required as it is freely available for clinical and research applications; however, proper citation is provided (Table 1) [11].

**Table 1:** Naranjo Adverse Drug Reaction Probability Scale Total Score Interpretation: ≥9 = Definite, 5-8 = Probable, 1-4 = Possible, ≤0 = Doubtful This scale is freely available for use [11].

Question	Yes	No	Do Not Know	Score
1. Are there previous conclusive reports on this reaction?	+1	0	0	
2. Did the adverse event appear after the suspected drug was administered?	+2	-1	0	
3. Did the adverse reaction improve when the drug was discontinued or a specific antagonist was administered?	+1	0	0	
4. Did the adverse reaction reappear when the drug was readministered?	+2	-1	0	
5. Are there alternative causes that could have caused the reaction?	-1	+2	0	
6. Did the reaction reappear when a placebo was given?	-1	+1	0	
7. Was the drug detected in blood or other fluids in concentrations known to be toxic?	+1	0	0	
8. Was the reaction more severe when the dose was increased or less severe when the dose was decreased?	+1	0	0	
9. Did the patient have a similar reaction to the same or similar drugs in any previous exposure?	+1	0	0	
10. Was the adverse event confirmed by objective evidence?	+1	0	0	

All identified ADRs were reported to the Pharmacovigilance Programme of India (PVPI) through the designated ADR monitoring centre at our institution. This reporting enhances international pharmacovigilance efforts and facilitates signal detection for rare ADRs.

Case identification protocol involved: (1) Systematic review of neurology clinic records for patients prescribed levetiracetam between January 2020-December 2024; (2) Screening of records for documentation of any new symptom developing after levetiracetam initiation; (3) Review by two independent neurologists to assess inclusion criteria; (4) Application of Naranjo scale to all potential cases; (5) Final inclusion only for cases with Naranjo score ≥5 and consensus agreement on unusual nature of ADR.

Statistical analysis

Data were analyzed using descriptive statistical methods. Continuous variables (age, levetiracetam dose) were summarized as means, standard deviations (SD), and ranges. Categorical variables (sex, adverse effect categories, management approaches) were summarized as frequencies and percentages (n, %). Due to the descriptive nature of this case series, no inferential statistical tests were applied. All analyses were performed using SPSS software (Version 26.0, 2019, IBM Corp., Armonk, NY, USA).

Ethical considerations

Ethical approval was obtained from the Institutional Human Ethics Committee of Mahatma Gandhi Medical College and Research Institute (approval number: MGMCRI/2026/04/IHEC/CS/03). Written informed consent for publication of anonymized case details was obtained from all patients or their legal guardians (for the 14-year-old pediatric patient). The study was conducted in accordance with the Declaration of Helsinki principles.

## Results

Fifteen cases met the inclusion criteria. The demographic and clinical characteristics are summarized in Table 2.

**Table 2:** List of uncommon adverse effects associated with levetiracetam (N=15) Data presented as original findings from the Mahatma Gandhi Medical College and Research Institute cohort (2020-2024). All patients provided informed consent for publication. ASM: Antiseizure medication; M: Male; F: Female. Naranjo scores: 5-8 = probable causality. Descriptive statistics only; no inferential tests applied.

Case	Age/Sex	Levetiracetam dose (mg/day)	Naranjo score	Adverse effect	Adverse effect category	Concomitant ASM
1	64/M	1000	7 (Probable)	Hypersexuality	Behavioural (n = 4, 27%)	Lacosamide
2	70/M	1500	6 (Probable)	Over-spirituality	Behavioural (n = 4, 27%)	Sodium valproate
3	14/M	500	8 (Probable)	Sudden decline in school performance	Cognitive (n = 3, 20%)	Phenytoin
4	25/F	1000	6 (Probable)	Excessive bleeding	Haematological (n = 1, 7%)	Phenytoin
5	46/M	1000	7 (Probable)	Suicidal tendency	Mood disorder (n = 3, 20%)	Brivaracetam
6	66/M	1500	5 (Probable)	Claustrophobia	Anxiety (n = 2, 13%)	Lacosamide
7	24/M	1500	7 (Probable)	Hypersexuality	Behavioural (n = 4, 27%)	Lacosamide
8	67/M	2000	6 (Probable)	Mania	Mood disorder (n = 3, 20%)	Sodium valproate
9	54/F	2000	5 (Probable)	Weight gain	Metabolic (n = 1, 7%)	Lacosamide
10	56/F	1500	7 (Probable)	Severe depression	Mood disorder (n = 3, 20%)	Brivaracetam
11	48/M	1500	6 (Probable)	Difficulty in urination	Genitourinary (n = 1, 7%)	Phenytoin
12	30/F	2000	7 (Probable)	Excessive daytime sleepiness	Cognitive (n = 3, 20%)	Phenytoin
13	36/M	1500	6 (Probable)	Change in mood and cognitive disturbances	Cognitive (n = 3, 20%)	Brivaracetam
14	30/F	1500	5 (Probable)	Insomnia	Anxiety (n = 2, 13%)	Sodium valproate
15	26/F	1500	7 (Probable)	Akathisia	Behavioural (n = 4, 27%)	Lacosamide

Demographics and dose

The cohort had a mean age of 43.1 years (SD ± 19.6 years; range: 8-70 years). The majority were male (9/15, 60%). The mean levetiracetam dose at the time of adverse effect reporting was 1400 mg/day (SD ± 450 mg/day; range: 500-2000 mg/day). No clear linear dose-response relationship was evident across all symptom types, with adverse effects occurring across the dosing spectrum.

Spectrum of adverse effects

Among the 15 patients, eight (53.3%) were on levetiracetam monotherapy, while seven (46.7%) received combination therapy with other ASMs, including phenytoin (n = 3), valproate (n = 2), oxcarbazepine (n = 1), and clobazam (n = 1). In combination therapy cases, the adverse effect either developed after levetiracetam initiation despite stable doses of concomitant ASMs or resolved following levetiracetam adjustment without changes to other medications, supporting levetiracetam's primary role.

**Figure 1:**
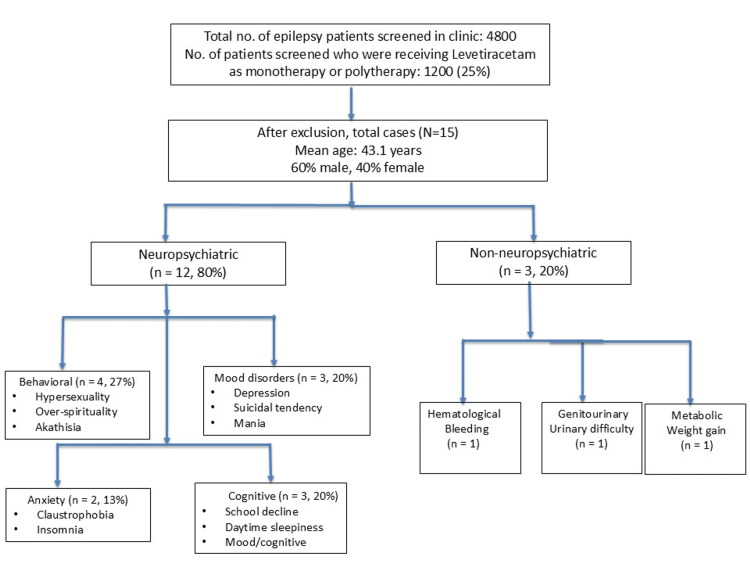
Spectrum of levetiracetam associated uncommon adverse effects (N=15) The distribution of uncommon levetiracetam-associated adverse effects among patients (n=15). Neuropsychiatric manifestations predominated (12/15, 80%) of all cases. Among these neuropsychiatric effects, behavioural dysregulation represented the largest subgroup (n = 4/15, 27%), followed by mood disorders (3/15, 20%) and cognitive disturbances (3/15, 20%), and anxiety-related symptoms accounting for 2/15 (13%). Non-neuropsychiatric effects constituted the remaining (3/15, 20%), including haematological, genitourinary, and metabolic complications. All cases demonstrated probable causality with Naranjo scores (n=15; range: 5 – 8). This figure represents original data from the Mahatma Gandhi Medical College and Research Institute cohort (2020-2024). ADR: Adverse drug reactions

Adverse effects were predominantly neuropsychiatric (12/15, 80%) (Figure 1). The neuropsychiatric manifestations observed included behavioural dysregulation comprising hypersexuality (n = 2), over-spirituality (n = 1), and akathisia (n = 1), accounting for 27% (n = 4) of all cases; mood disorders including severe depression (n = 1), suicidal tendency (n = 1), and mania (n = 1), representing 20% (n = 3) of the cohort; anxiety and perception disturbances such as claustrophobia (n = 1) and insomnia (n = 1), comprising 13% (n = 2); and cognitive disturbances including sudden decline in school performance (n = 1), unusual excessive daytime sleepiness (n = 1), and mood changes with cognitive disturbances (n = 1), also accounting for 20% (n = 3) of cases. The remaining 20% (n = 3) consisted of non-neuropsychiatric adverse effects, which included excessive bleeding manifesting as epistaxis and gingival bleeding (n = 1), difficulty in urination (n = 1), and significant weight gain of 8 kg over three months (n = 1).

Temporal relationship and management

The mean time from levetiracetam initiation or dose adjustment to adverse effect onset was 30 days (range: 7-90 days). Table 3 summarizes the management strategies and outcomes for all patients.

**Table 3:** Management strategies and outcomes for levetiracetam-associated adverse effects (N = 15) Adjunctive psychiatric pharmacotherapy included selective serotonin reuptake inhibitors for depression, antipsychotics for mania, and benzodiazepines for anxiety symptoms. ASM: Antiseizure medication.

Management parameter	Category	Number of patients (N=15)	Percentage (%)
Primary intervention	Dose reduction	4	27%
	Switch to alternative ASM	11	73%
Alternative ASM selected	Lacosamide	5	33%
	Sodium valproate	3	20%
	Brivaracetam	3	20%
Adjunctive psychiatric consultation	Required	5	33%
	Not required	10	67%
Time to symptom resolution	1-3 months	8	53%
	4-6 months	7	47%
Mean time to complete resolution	3-4 months (Range: 1-6 months)		

Dechallenge and rechallenge outcomes

Dechallenge (medication discontinuation or dose reduction) was performed in all 15 patients. All patients (100%) demonstrated positive dechallenge, defined as partial or complete resolution of the adverse effect following levetiracetam dose reduction or withdrawal. Complete resolution occurred in 13 patients (86.7%), while partial improvement (defined as >50% reduction in symptom severity per clinical assessment) occurred in two patients (13.3%).

Rechallenge (re-exposure to levetiracetam) occurred inadvertently in two patients (13.3%) who restarted levetiracetam due to seizure recurrence on alternative ASMs. Both patients experienced recurrence of the same adverse effect (hypersexuality in one, severe depression in the other) within two to four weeks of rechallenge, confirming positive rechallenge. The remaining 13 patients were not rechallenged due to ethical considerations and the availability of effective alternative therapy.

## Discussion

In our study, we described 15 patients who developed uncommon adverse effects during levetiracetam therapy, contributing to the growing body of literature on the diverse neuropsychiatric and non-neuropsychiatric manifestations associated with this widely used ASM. The evidence from this series supports that levetiracetam can lead to a variety of clinically significant adverse events beyond the well-characterized somnolence, dizziness, and irritability.

Spectrum of adverse effects

The predominance of neuropsychiatric adverse events (80%) in our cohort aligns with previous reports highlighting levetiracetam's psychotropic potential [12]. However, the specific manifestations observed, including hypersexuality (n = 2), marked over-spirituality (n = 1), and akathisia (n = 1), represent particularly unusual presentations with limited prior documentation. Hypersexuality has been rarely reported with levetiracetam, with proposed mechanisms involving dopaminergic modulation in mesolimbic pathways or disinhibition of frontal lobe-mediated impulse control [13]. The case of over-spirituality (intensified religious ideation and behaviour) is exceptionally rare and may represent a culturally mediated manifestation of frontal lobe dysfunction or mood dysregulation. Levetiracetam-related neurobehavior dysfunction is primarily attributed to the modulation of the AMPA receptor [14].

The cognitive disturbances observed, including a sudden decline in school performance in the 14-year-old and subjective cognitive complaints in the adults, warrant particular attention. While levetiracetam is generally considered cognitively neutral compared to older ASMs, emerging evidence suggests a subset of patients may experience dose-dependent cognitive slowing, particularly affecting verbal fluency and processing speed [15]. Paediatric patients may be especially vulnerable to academic decline, necessitating close monitoring of school performance after treatment initiation.

Non-neuropsychiatric adverse effects, while less frequent (20%), raise important mechanistic considerations. The case of excessive bleeding (epistaxis, gingival bleeding) warrants detailed mechanistic consideration. SV2A is not only present in the brain but also in platelet dense granules, where it plays a role in calcium-dependent granule exocytosis and platelet activation [16]. Modulation of platelet SV2A by levetiracetam could theoretically impair platelet function by reducing the release of pro-aggregatory factors such as ADP and serotonin. Additionally, the concomitant administration of phenytoin in this patient introduces a second potential mechanism: phenytoin is known to induce hepatic cytochrome P450 enzymes, accelerating vitamin K metabolism and potentially reducing the synthesis of vitamin K-dependent clotting factors (II, VII, IX, X). The combination of impaired platelet function (from levetiracetam) and reduced coagulation factor synthesis (from phenytoin) could produce a synergistic bleeding risk. However, given the rarity of such reports and idiosyncratic reactions, undiagnosed inherited bleeding disorders, or alternative explanations cannot be excluded.

Dose relationships and individual susceptibility

The lack of a clear dose-dependency in our series (adverse effects occurring at doses ranging from 500-2000 mg/day) suggests individual susceptibility factors may play a more critical role than cumulative drug exposure. This observation aligns with pharmacogenetic studies suggesting polymorphisms in the SV2A gene (SV2A rs1416309) or genes encoding drug transporters may influence levetiracetam neuropsychiatric toxicity [17]. Additionally, variations in dopamine receptor genes (DRD2, DRD3) have been associated with behavioural adverse effects, supporting a gene-environment interaction model [18].

The mean onset of 30 days (range 7-90 days) following drug initiation or dose adjustment represents a clinically actionable finding. This temporal pattern suggests that the first three months of therapy constitute a critical monitoring period during which clinicians should maintain heightened vigilance for emerging neuropsychiatric or physical symptoms. Early recognition within this window allows for prompt intervention, either dose reduction or medication switch, potentially preventing symptom progression and improving patient adherence. This timeframe also aligns with typical post-prescription follow-up schedules, supporting the practice of scheduling early review visits within four to six weeks of levetiracetam initiation to specifically assess for unusual adverse effects.

Gender distribution

We observed a male predominance in our cohort (60%), which contrasts with some studies suggesting women may exhibit higher rates of levetiracetam-induced behavioral manifestations [19]. Cramer et al. reported that women with epilepsy may be more susceptible to mood-altering effects of ASMs, potentially due to hormonal influences on neurotransmitter systems and drug metabolism [19]. However, other large cohort studies have demonstrated no significant gender differences in psychiatric ADR rates [20]. Our male predominance likely reflects the small sample size and potential sampling bias inherent in a single-centre retrospective study, rather than a true gender-based risk difference. The higher proportion of male patients in our epilepsy clinic population (approximately 55% overall) may also contribute to this observation.

Mechanistic insights

Levetiracetam's unique mechanism - binding to SV2A - distinguishes it from other ASMs and may explain both its efficacy and its unusual adverse effect profile. SV2A is ubiquitously expressed in synaptic vesicles throughout the central nervous system, modulating calcium-dependent exocytosis of neurotransmitters [3]. In addition to its role in inhibitory and excitatory neurotransmitter release, SV2A is expressed in neuroendocrine tissues, platelets, and pancreatic beta-cells, providing plausible mechanisms for non-neuropsychiatric adverse effects such as bleeding (platelet SV2A) and weight gain or metabolic disturbances (pancreatic SV2A involvement in insulin secretion). The mechanism of bleeding is supported by studies demonstrating SV2A expression in megakaryocytes and its involvement in vesicle trafficking during thrombopoiesis [21].

The predominance of neuropsychiatric effects likely reflects SV2A's critical role in regulating monoamine neurotransmitter systems, particularly dopamine, serotonin, and norepinephrine. Positron emission tomography studies have demonstrated that therapeutic levetiracetam concentrations occupy 50-80% of brain SV2A sites, with inter-individual variability in occupancy potentially explaining differential susceptibility to adverse effects [22].

Levetiracetam prescribing should include clear pre-treatment counselling about potential psychiatric and non-neuropsychiatric adverse effects, with emphasis on early reporting and stigma reduction. Close monitoring during the first three to six months is essential, particularly after dose changes. If unusual effects arise, clinicians should individualize management through dose adjustment, switching therapy, and early multidisciplinary involvement when needed.

Strengths of the study

Strengths of this study include systematic case identification using standardized inclusion criteria, application of Naranjo causality assessment by two independent reviewers, comprehensive reporting of dechallenge/rechallenge outcomes, contribution to pharmacovigilance through PVPI reporting, and description of other manifestations like over-sprituality which were rarely reported in Western literature.

Limitations of the study

This study has several limitations that must be considered when interpreting the findings. First, its retrospective design introduces potential information and recall bias; second, the single-centre setting and small sample size (n=15) limit the generalizability of our findings and the ability to perform robust statistical analyses to identify risk factors. Lack of standardized psychiatric assessment scales. The presence of concomitant ASMs in 47% of patients complicates definitive attribution of adverse effects solely to levetiracetam. However, temporal relationships (onset after levetiracetam introduction, resolution after levetiracetam adjustment without changing other medications) support a causal role. Future prospective studies should employ standardized assessment tools to evaluate potential drug-drug interactions and synergistic neuropsychiatric effects. Fourth, we could not estimate the true incidence of these uncommon effects, as this was not an aim of the study design. There were no genetic or pharmacokinetic data to explore individual susceptibility factors.

Future directions

Future research should focus on large, prospective, multi-centre registries to accurately determine the incidence of these uncommon adverse effects. Such studies should incorporate standardised psychiatric and non-neuropsychiatric adverse effect assessments and collect data on potential genetic and pharmacokinetic risk factors to identify patients at higher risk. This would allow for more personalised counselling and monitoring strategies.

The clinical relevance of our study can be summarized in the following points. These findings highlight the need for thorough prospective counselling of patients and their caregivers regarding potential adverse effects before starting levetiracetam. Physicians need to exercise heightened awareness and vigilance for new psychiatric or non-neuropsychiatric symptoms occurring after the initiation of levetiracetam therapy. As demonstrated, early detection of symptom change will demand a medication change, but also provides the basis for managing patients and will usually prolong the time to recovery.

## Conclusions

This study describes 15 patients who developed unusual neuropsychiatric and physical adverse effects during levetiracetam therapy, including rarely reported manifestations such as hypersexuality, over-spirituality, and bleeding. The mean onset of 30 days (range 7-90 days) provides a clinically useful timeframe for targeted monitoring, enabling earlier recognition and intervention to optimize patient outcomes. The consistent temporal relationship - onset following drug initiation, improvement after dose reduction or withdrawal, and recurrence upon rechallenge in two patients - supports a causal association, with neuropsychiatric effects predominating (80% of cases). These findings underscore that while levetiracetam remains a cornerstone of epilepsy treatment, individual susceptibility rather than dose alone may precipitate uncommon but severe reactions, highlighting the need for routine monitoring and proactive counselling, particularly during the first three months of therapy.

As a descriptive series, this study cannot determine true incidence or definitive risk factors; however, these observations should alert clinicians to atypical presentations and generate hypotheses for future research. Large, prospective, multi-center registries incorporating standardized psychiatric assessments and genetic analyses are needed to better define the mechanisms and risk factors underlying these distressing reactions to optimize patient safety and quality of life.

## References

[1] Brodie MJ, Perucca E, Ryvlin P, Ben-Menachem E, Meencke HJ (2007). Comparison of levetiracetam and controlled-release carbamazepine in newly diagnosed epilepsy. Neurology.

[2] Patsalos PN (2004). Clinical pharmacokinetics of levetiracetam. Clin Pharmacokinet.

[3] Lynch BA, Lambeng N, Nocka K, Kensel-Hammes P, Bajjalieh SM, Matagne A, Fuks B (2004). The synaptic vesicle protein SV2A is the binding site for the antiepileptic drug levetiracetam. Proc Natl Acad Sci U S A.

[4] Kaminski RM, Matagne A, Leclercq K (2008). SV2A protein is a broad-spectrum anticonvulsant target: functional correlation between protein binding and seizure protection in models of both partial and generalized epilepsy. Neuropharmacology.

[5] Mula M, Trimble MR, Sander JW (2004). Psychiatric adverse events in patients with epilepsy and learning disabilities taking levetiracetam. Seizure.

[6] Halma E, de Louw AJ, Klinkenberg S, Aldenkamp AP, IJff DM, Majoie M (2014). Behavioral side-effects of levetiracetam in children with epilepsy: a systematic review. Seizure.

[7] Helmstaedter C, Fritz NE, Kockelmann E, Kosanetzky N, Elger CE (2008). Positive and negative psychotropic effects of levetiracetam. Epilepsy Behav.

[8] Oghlakian R, Nock C, Koubeissi M (2010). A case of levetiracetam-induced thrombocytopenia. Epileptic Disord.

[9] Josephson CB, Engbers JD, Jette N (2019). Prediction tools for psychiatric adverse effects after levetiracetam prescription. JAMA Neurol.

[10] Steinhoff BJ, Staack AM (2019). Levetiracetam and brivaracetam: a review of evidence from clinical trials and clinical experience. Ther Adv Neurol Disord.

[11] Naranjo CA, Busto U, Sellers EM (1981). A method for estimating the probability of adverse drug reactions. Clin Pharmacol Ther.

[12] Ettinger AB, Kustra RP, Hammer AE (2007). Effect of lamotrigine on depressive symptoms in adult patients with epilepsy. Epilepsy Behav.

[13] Calabrò RS, Marino S, Bramanti P (2011). Sexual and reproductive dysfunction associated with antiepileptic drug use in men with epilepsy. Expert Rev Neurother.

[14] Carunchio I, Pieri M, Ciotti MT, Albo F, Zona C (2007). Modulation of AMPA receptors in cultured cortical neurons induced by the antiepileptic drug levetiracetam. Epilepsia.

[15] Witt JA, Helmstaedter C (2012). Should cognition be screened in new-onset epilepsies? A study in 247 untreated patients. J Neurol.

[16] Bajjalieh SM, Frantz GD, Weimann JM, McConnell SK, Scheller RH (1994). Differential expression of synaptic vesicle protein 2 (SV2) isoforms. J Neurosci.

[17] Lynch BA, Matagne A, Brännström A, von Euler A, Jansson M, Hauzenberger E, Söderhäll JA (2008). Visualization of SV2A conformations in situ by the use of protein tomography. Biochem Biophys Res Commun.

[18] Zhang JF, Piryani R, Swayampakula AK, Farooq O (2022). Levetiracetam-induced aggression and acute behavioral changes: a case report and literature review. Clin Case Rep.

[19] Cramer JA, Arrigo C, Van Hammée G, Gauer LJ, Cereghino JJ (2000). Effect of levetiracetam on epilepsy-related quality of life. Epilepsia.

[20] Mula M, Sander JW (2007). Suicidal ideation in epilepsy and levetiracetam therapy. Epilepsy Behav.

[21] Xu T, Bajjalieh SM (2001). SV2 modulates the size of the readily releasable pool of secretory vesicles. Nat Cell Biol.

[22] Finnema SJ, Rossano S, Naganawa M (2019). A single-center, open-label positron emission tomography study to evaluate brivaracetam and levetiracetam synaptic vesicle glycoprotein 2A binding in healthy volunteers. Epilepsia.

